# Molecular Recognition of Agonist and Antagonist for Peroxisome Proliferator-Activated Receptor-α Studied by Molecular Dynamics Simulations

**DOI:** 10.3390/ijms15058743

**Published:** 2014-05-15

**Authors:** Mengyuan Liu, Lushan Wang, Xian Zhao, Xun Sun

**Affiliations:** 1 State Key Laboratory of Crystal Materials, Shandong University, 27 Shanda Nanlu, Jinan 250100, China; E-Mail: lmyiop@sina.com; 2 State Key Laboratory of Microbial Technology, Shandong University, 27 Shanda Nanlu, Jinan 250100, China; E-Mail: lswang@sdu.edu.cn

**Keywords:** PPAR-α, molecular modeling, diabetes

## Abstract

Peroxisome proliferator activated receptor-α (PPAR-α) is a ligand-activated transcription factor which plays important roles in lipid and glucose metabolism. The aim of this work is to find residues which selectively recognize PPAR-α agonists and antagonists. To achieve this aim, PPAR-α/13M and PPAR-α/471 complexes were subjected to perform molecular dynamics simulations. This research suggests that several key residues only participate in agonist recognition, while some other key residues only contribute to antagonist recognition. It is hoped that such work is useful for medicinal chemists to design novel PPAR-α agonists and antagonists.

## 1. Introduction

**Figure 1.**
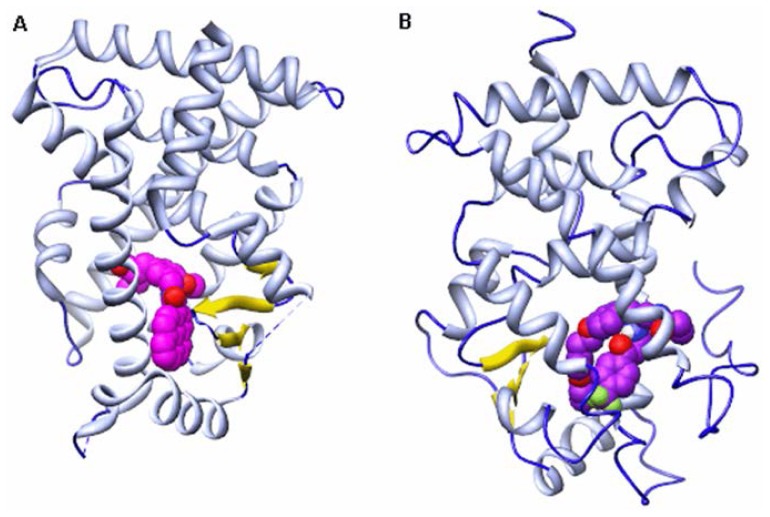
The overall structures of peroxisome proliferator activated receptor-α (PPAR-α)/ligand complexes. (**A**) PPAR-α/13M complex; and (**B**) PPAR-α/471 complex. PPAR-α backbone is shown in ribbon (Helix: white; Strand: yellow; Coil: blue). Agonist and antagonist are shown in sphere (Carbon atom: purple; Oxygen atom: red; Nitrogen atom: blue; Fluorine atom: green).

**Figure 2.**
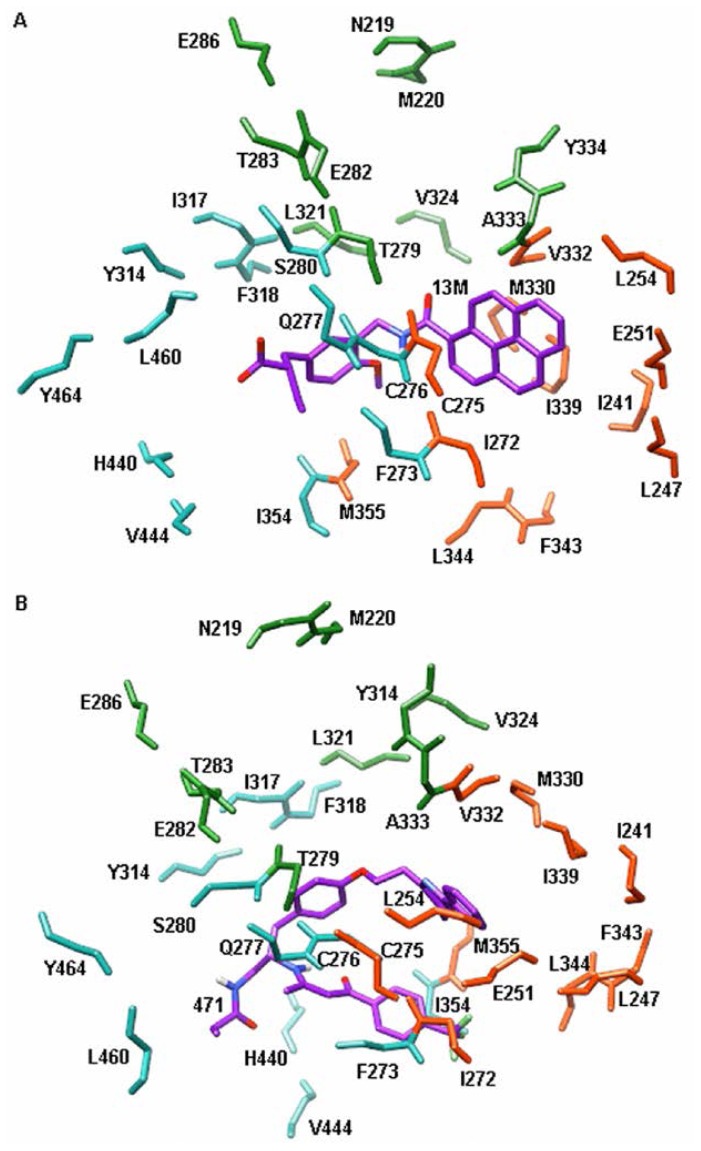
The active site of PPAR-α. (**A**) PPAR-α/13M complex; and (**B**) PPAR-α/471 complex. Residues in PPAR-α are only shown with backbone atoms. Agonist and antagonist are shown in stick with purple carbon atoms. The arm I region is shown in stick with blue atoms. The arm II region is shown in stick with orange atoms. The entrance region is shown in stick with green atoms. For the sake of clarity, only the polar hydrogen atoms are displayed.

Peroxisome proliferator activated receptors (PPARs) are DNA-binding transcription factors belonging to the nuclear hormone receptor super family [1–4]. To date, three distinct PPAR subtypes have been identified namely PPAR-α, PPAR-β (also known as PPAR-δ) and PPAR-γ [5–7]. Among these subtypes, PPAR-α is a key regulator of lipid and glucose metabolism. Activation of PPAR-α can increase high density lipoprotein, decrease triglycerides, increase insulin sensitivity and reduce adiposity. Therefore, it becomes an attractive target for treating type II diabetes and its complications [8–12]. Due to this reason, the structures of PPAR-α have been intensively studied at the atomic level in recent years and several X-ray crystal structures of PPAR-α have been determined (Figure 1). The results suggest that the active site of PPAR-α consists of three parts: arm I, arm II and entrance regions [13] (Figure 2).

Based on the obtained crystal structures, lots of researches have been conducted on PPAR-α agonists [14–16]. Besides agonists, the antagonists are also useful because of the need for fully understanding the pharmacology of PPAR-α. Thus, new research efforts have been made to explore the potential utility of PPAR-α antagonists [1]. And several PPAR-α antagonists have been reported [17–19].

**Figure 3.**
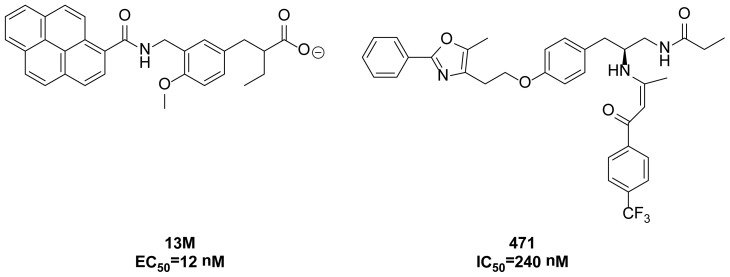
Chemical structures of PPAR-α agonist 13M and antagonist 471.

In order to develop more potent PPAR-α agonists and antagonists, it is necessary to find key residues which only contribute to agonist (or antagonist) recognition. Previous research suggests that agonists form polar interactions with S280, Y314, H440 and Y464, which are responsible for agonist recognition [13]. Besides these residues, other polar residues in the binding pocket can also form strong polar interactions with ligands and participate in agonist (or antagonist) recognition. Considering that apolar interactions are crucial for molecular recognition, we deduce that some hydrophobic residues also play important roles in agonist (or antagonist) recognition. Thus, the aim of this work is to find whether some other residues can be involved in agonist (or antagonist) recognition. So far, the systematic researches on this issue are limited, which may hinder rational design of more potent PPAR-α agonists and antagonists. To achieve this goal, the researchers must determine the interaction strength between ligands and residues in PPAR-α, which cannot be compared by only inspecting the crystal structures. Under this condition, molecular dynamics simulation is a useful tool to achieve this goal. Thus, conventional molecular dynamics simulations of PPAR-α in complex with an agonist 13M, as well as an antagonist 471 were performed (Figure 3). It is hoped that these findings can provide useful information to help medicinal chemists design more potent PPAR-α agonists and antagonists.

## 2. Results and Discussion

### 2.1. Backbone Stability

**Figure 4.**
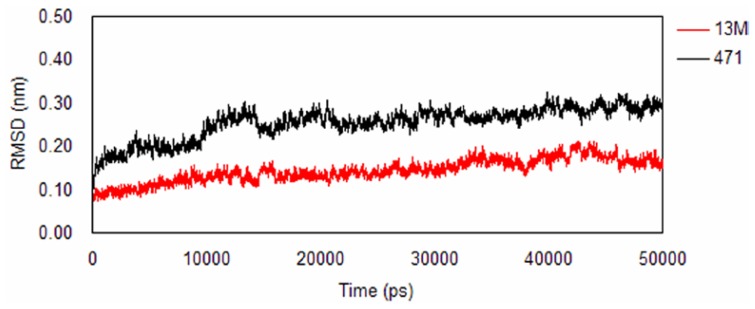
The root mean square deviation (RMSD) of Cα atoms for different systems. 13M: PPAR-α/13M complex; 471: PPAR-α/471 complex.

The root mean square deviation (RMSD) for backbone Cα atoms respect to initial structures of production dynamics was calculated. It can be observed form Figure 4 that the RMSD values for PPAR-α/13M complex fluctuate around 0.1 nm in the period of 20–50 ns, while the values for PPAR-α/471 complex stabilize at about 0.25 nm. These results indicate that both systems reach equilibrium within 20 ns and the trajectories of the last 30 ns can be used to perform hydrogen bond and energy decomposition analyses.

### 2.2. Hydrogen Bond Analysis

**Figure 5.**
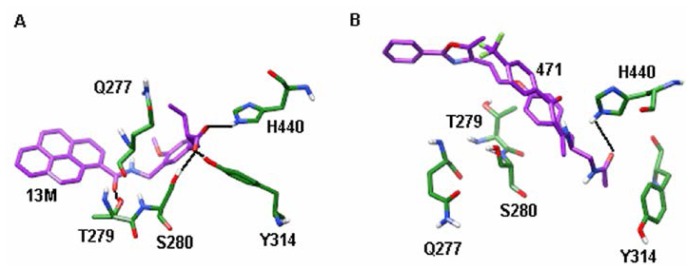
Snapshots of PPAR-α/ligand complexes at 50 ns (**A**) PPAR-α/13M complex; and (**B**) PPAR-α/471 complex. Agonist and antagonist are shown in stick with purple carbon atoms, while residues of PPAR-α are shown in stick with green carbon atoms. The hydrogen bonds are shown in black lines (For the sake of clarity, only the polar hydrogen atoms are displayed).

Stable hydrogen bonds are crucial for molecular recognition. Residues which form more stable hydrogen bonds with agonist than with antagonist will be considered to only participate in agonist recognition. On the contrary, residues which form more stable hydrogen bonds with antagonist than with agonist will be considered to only participate in antagonist recognition. Considering that the hydrogen bond stability cannot be compared by inspecting the crystal structures, molecular dynamics simulations must be used. Table 1 lists the hydrogen bond probability of ligands with some residues in PPAR-α. It can be seen that all of the five residues form more stable hydrogen bonds with 13M than 471, which suggests that Q277, T279, S280, Y314 and H440 only take part in PPAR-α agonist recognition. Among these hydrogen bonds, the hydrogen bonds of 13M with Q277 and T279 (Figure 5A) cannot be seen from the crystal structure. Based on this finding, we advise medicinal chemists to make designed PPAR-α agonists form hydrogen bonds with Q277 and T279. However, it must be noted that the hydrogen bond stability is not enough to determine which residues can differentiate agonist and antagonist because this analysis only in some extent reflect the electrostatic interactions. To more fully explore the interaction strength of residues with agonists and antagonists, the interaction energies must be calculated.

### 2.3. Energy Decomposition Analysis

**Figure 6.**
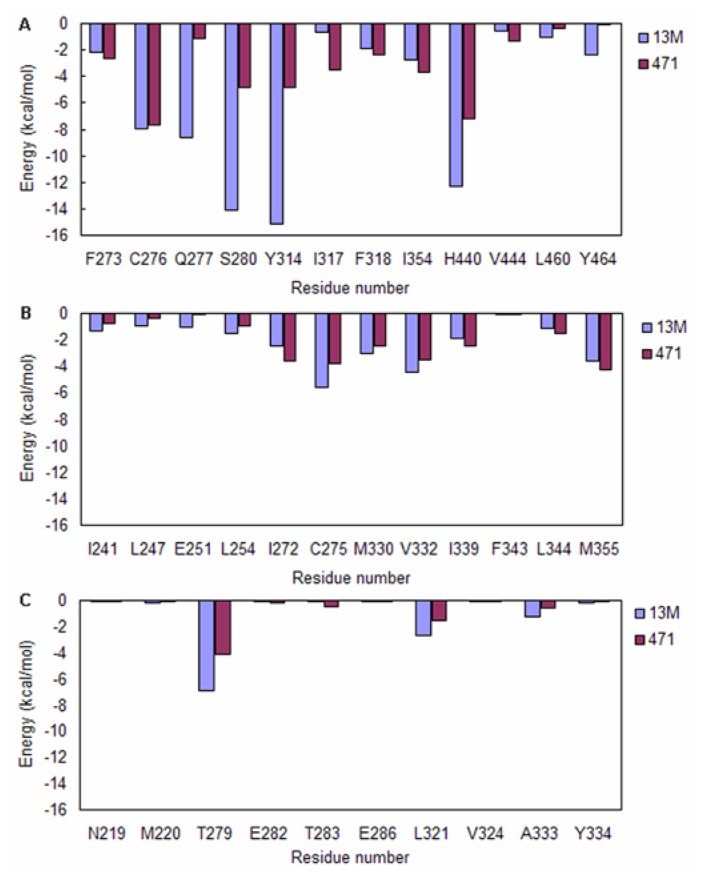
The average total interaction energies of agonist and antagonist with residues in (**A**) Arm I region; (**B**) Arm II region; and (**C**) Entrance region.

Considering that both of the 13M and 471 occupy the arm I, arm II and entrance region, the interaction energies of residues in these regions with 13M and 471 cannot be judged by inspecting the crystal structures. So the energy decomposition analysis must be carried out. The calculated results are shown in Figure 6. Residues which only exhibit strong interactions with 13M will be considered to have selectivity for agonist recognition, while residues which only exhibit strong interactions with 471 will be considered to have selectivity for antagonist recognition.

In arm I region, the interaction energies of 13M with Q277, S280, Y314, H440 and Y464 are stronger than 471 (Figure 6A), which indicates that these residues selectively recognize agonists. In contrast, 471 binds I317 and I354 more tightly than 13M (Figure 6A). This suggests that the two residues can selectively recognize antagonists. The previous researches have reported that S280, Y314, H440 and Y464 are responsible for agonist recognition [13], which are consistent with our studies.

In arm II region, the interactions of 13M with C275 and V332 are much stronger than 471, indicating that the two residues only make contributions to agonist recognition (Figure 6B). Unlike C275 and V332, the non-bonded interactions between 471 and I272 are stronger than 13M, which suggests that this residue can only be responsible for antagonist recognition (Figure 6B).

In entrance region, the difference in interaction energies of ligands with T279 and L321 is significant (Figure 6C). 13M binds these two residues more tight than 471. Therefore, it can be inferred that T279 and L321 only contribute to agonist recognition.

## 3. Experimental Section

### 3.1. System Preparation

The X-ray crystal structures of PPAR-α/13M (PDB code: 3VI8) [20] and PPAR-α/471 (PDB code: 1KKQ) [21] complexes were obtained from the RCSB Protein Data Bank. Crystal water molecules within 4 Å of ligands were kept. For PPAR-α/471 complex, only the A chain was kept. And the co-repressor in PPAR-α/471 complex was also removed because this work is only to explore the interactions of PPAR-α with ligands. Finally, the hydrogen atoms were added by Maestro (Schrodinger LLC, New York, NY, USA).

### 3.2. Molecular Dynamics Simulations

Molecular dynamics simulations were performed using Gromacs 4.5.3 program [22–25]. The force field for proteins was Amber FF99SB [26,27], while for agonist and antagonist was General Amber Force Field (GAFF) [28]. The systems were immersed in a SPC (simple point charge) water [29] box of 1.0 nm from the solute surface. The sodium ions were then added to neutralize the systems. The Particle Mesh Ewald (PME) method [30–32] was used for correcting electrostatic interaction. The LINCS algorithm [33,34] was employed to constrain all bonds involving hydrogen atoms. Periodic boundary conditions were also used. The non-bonded cutoff distance was set to 1.0 nm. The temperature was kept at 300 K with V-rescale temperature coupling [35]. The time step was 1.0 fs. The trajectories were sampled every 10 ps in molecular dynamics simulations. Steepest descent energy minimization was first performed to give the maximum force below 1000 kJ·mol^−1^·nm^−2^ in order to remove the steric clash. After that, the complexes were then equilibrated by 100 ps position restraint MD simulations with 1000 kJ·mol^−1^·nm^−2^ constant force on the heavy atoms of proteins and ligands under NVT (constant number of molecules, volume and temperature) condition. The X-ray crystal structure of PPAR-α/13M complex missed residues 231–237 and 263–264. But these residues are far away from the binding sites of agonist 13M. Therefore, the impact of the missing residues on agonist binding is limited. Considering this, 1 ns equilibrium simulation and 50 ns production run with restraints on the Cα atoms of residues 230, 238, 262 and 265 (restraint force constant = 1000 kJ·mol^−1^·nm^−2^) were sequentially carried out under NVT condition. Unlike PPAR-α/13M complex, no missing residue was found in PPAR-α/471 complex. So no position restraints were applied in the above 1 ns equilibrium simulation and 50 ns production run under NVT condition.

### 3.3. Hydrogen Bond Analysis

To define the presence of hydrogen bond, an acceptor–donor distance within 0.35 nm, and an acceptor–hydrogen–donor angle within 30° was used [36]. The probability of hydrogen bond was calculated using the following equation [37]:

(1) $$P_{\text{hbond}}=\frac{N_{\text{existence}}}{N_{\text{total}}}\times 100\%$$

where *P*_hbond_ was the probability of hydrogen bond. *N*_existence_ was the number of frames that investigated hydrogen bonds existed. *N*_total_ was the total number of collected frames in production phase. The probability of each hydrogen bond was calculated in terms of a percentage that varied from 0% to 100%, where a percentage of 100 indicated that the hydrogen bond was highly stable and a percentage of 0 indicated an unstable hydrogen bond. Three thousand snapshots isolated from the last 30 ns production runs with an interval of 10 ps were employed for hydrogen bond analysis.

### 3.4. Energy Decomposition Analysis

The electrostatic (*E*_elec_) and van der Waals (*E*_vdw_) interaction energies of some residues in PPAR-α with ligands were calculated according to the Amber force field equation. The total interaction energies between residues in PPAR-α with ligands are the sum of *E*_elec_ and *E*_vdw_. All energy components are calculated using the same snapshots as the hydrogen bond analysis.

## 4. Conclusions

In conclusion, the hydrogen bond and energy decomposition analyses suggest that S280, Y314, H440 and Y464 only participate in agonist recognition, which is accord with the previous reports [13]. What is more, our research suggests that C275, Q277, T279, L321 and V332 are only involved in agonist recognition, while I272, I317 and I354 only contribute to antagonist recognition. It is advised that medicinal chemists can make strong non-bonded interactions (such as hydrogen bonds, salt bridges and π–π stacking interactions) with the above residues when they design PPAR-α agonists and antagonists.

## Figures and Tables

**Table 1.** The probability (%) of hydrogen bonds between PPAR-α and ligands.

Residues	Ligands	

	13M	471
Q277	31.1	0.0
T279	78.5	0.1
S280	99.7	19.1
Y314	100.0	0.0
H440	88.8	73.3
